# Migrant women’s engagement in health-promotive activities through a women’s health collaboration

**DOI:** 10.3389/fpubh.2023.1106972

**Published:** 2023-06-15

**Authors:** Cecilia Lindsjö, Katarina Sjögren Forss, Christine Kumlien, Anders Kottorp, Margareta Rämgård

**Affiliations:** Department of Care Science, Faculty of Health and Society, Malmö University, Malmö, Sweden

**Keywords:** health equality, community-based participatory research, health literacy, health promotion, migrant women, social support, story-dialog method, lay health promoter

## Abstract

**Introduction:**

Social determinants of health impact health, and migrants are exposed to an inequitable distribution of resources that may impact their health negatively, leading to health inequality and social injustice. Migrant women are difficult to engage in health-promotional activities because of language barriers, socioeconomic circumstances, and other social determinants. Based on the framework of Paulo Freire, a community health promotion program was established in a community-academic partnership with a community-based participatory research approach.

**Aim:**

The aim of this study was to describe how a collaborative women’s health initiative contributed to migrant women’s engagement in health promotion activities.

**Materials and methods:**

This study was part of a larger program, carried out in a disadvantaged city area in Sweden. It had a qualitative design with a participatory approach, following up on actions taken to promote health. Health-promotional activities were developed in collaboration with a women’s health group, facilitated by a lay health promoter. The study population was formed by 17 mainly Middle Eastern migrant women. Data was collected using the story-dialog method and the material was analyzed using thematic analysis.

**Result:**

Three important contributors to engagement in health promotion were identified at an early stage of the analysis process, namely, the group forming a social network, the local facilitator from the community, and the use of social places close to home. Later in the analysis process, a connection was made between these contributors and the rationale behind their importance, that is, how they motivated and supported the women and how the dialog was conducted. This therefore became the designated themes and were connected to all contributors, forming three main themes and nine sub-themes.

**Conclusion:**

The key implication was that the women made use of their health knowledge and put it into practice. Thus, a progression from functional health literacy to a level of critical health literacy may be said to have occurred.

## Introduction

Health is formed by social determinants of health, influencing one’s resources to handle one’s own health (1). Health inequalities can be ascribed to the inequitable distribution of power, income, and other determinants of health (1). During transit and post-migration, many adverse experiences and situations may occur, influencing health negatively (2). Poor socioeconomic status, social isolation, or exclusion from the host society may be a reality experienced by migrant populations (3). Accordingly, one may argue that migrants are exposed to an inequitable distribution of resources that may impact their health negatively, leading to health inequity. Promoting migrant health should therefore be prioritized.

Health promotion is about creating or enhancing conditions conducive to health (4). According to the World Health Organization (WHO), the definition of health promotion includes working for people’s own control over their health (4). It has also been suggested that the concept of health literacy is useful in the context of health promotion, since it focuses on factors contributing to health inequity which can be used for the health-promotive work (5).

*“Health literacy is linked to literacy and entails people’s knowledge, motivation and competences to access, understand, appraise, and apply health information in order to make judgments and take decisions in everyday life concerning healthcare, disease prevention and health promotion to maintain or improve quality of life during the life course.”* (6).

Hence, the concept of health literacy is related to health promotion (7). However, health promotion among migrant women has proved challenging; for example, health care has had difficulties achieving healthy lifestyle changes within the population (8). Previous research has tried to find answers to this in the definitions of health and lifestyle within the population itself (8). It showed, for example, that the identity of being Moroccan, Muslim, and mother was stronger than the concept of health and lifestyle, making family or cultural norms more prioritized than individual health (8). Besides, physical activity (PA) barriers among migrants in the US have previously been identified as having work indoors, lacking financial resources for PA fees, and lack of transport or language barriers (9). Household work, taking care of children, and not being culturally comfortable were also reasons to refrain from PA (9). Taking a first step to PA was difficult due to being unacquainted with PA environments (9). Moreover, for migrants in a socially disadvantaged neighborhood, the concept of lifestyle included competing values (10). An example was that healthy eating habits could put social relationships at risk (10). Thus, health behavior may not be determined wholly by the individual, but by the collective social practice (10). In Sweden too women migrants have difficulties engaging in PA (11). Previous research in a Swedish context has promoted health information to migrants, which has proved successful (12–14), but the delivery of the information needs to be further modified, and factors such as age and health literacy should be considered (13, 14). Thus, further recognition of the importance of establishing health promotion activities among migrant women may be needed.

The migrant population is, as any native population, heterogeneous, regarding health, health needs, and health promotion. However, for migrants’ other measures for health promotion may be eligible and other components may be needed, besides education or information, to assist them to engage in PA (9, 12). Combining education with components focusing on barriers, and including the perspectives of the migrant population, may be useful (9, 12). One study recommends considering participant perspectives on health promotion when forming interventions, as well as including social aspects and not only focusing on an individual perspective (8). In a socially disadvantaged neighborhood, the combination of healthy behavior with social life was preferred by a migrant population (10). The mode of information delivery may also be relevant to consider (15). Among a population of migrant women who received health information, interactive talks or presentations were preferred (15). This can be related to the pedagogy of Paulo Freire where the teaching starts from the student’s situation, using dialog (16). Freire’s idea of dialog included reflection and action by the community, alongside more traditional aspects, such as talk and discussion (17). His goal was to create more social justice and empowerment for marginalized people using participatory dialogs. Freire therefore developed a pedagogy for marginalized populations and believed that the emancipation of oppressed populations was best dealt with through teaching critical consciousness (16). The Freirean pedagogy, together with action research, initiated by Lewin, forms a base for participatory action research (17). The community-based participatory research (CBPR) approach was then developed based on these theories on dialog and empowerment (18). Thus, within the CBPR approach, dialog and participant perspectives are in focus. This approach was therefore used in the Collaborative Innovations for Health Promotion program within which this study was conducted.

There are some core principles and values in participatory research. One such principle is to “maximize participation” (18). The participatory research approach acknowledges that there are many truths to an issue or a phenomenon that is under study, which can be enriching and give a nuanced perspective (19). When conducting participatory research one aims to involve people or communities, affected by the research, in the research process, thus considering the study population’s perspective along the way in a bottom-up approach (17). Another principle is “to enable people to take action,” that is, researchers should facilitate for people to take action in an empowering way (18). Everyone’s knowledge is valuable in participatory research and useful in the process of change. Thus, the hierarchy of knowledge should be set aside, which may also influence relations between participants (18). A third principle is to work for sustainability by adding local value (18). The phenomenon being studied should consequently be relevant to the local community (18). In line with this principle, a previous study points to how participatory research has been successfully used in various health intervention studies that were relevant and met the needs of the population (19). So, based on the needs expressed by the women, a women’s health group was formed in the CBPR program Collaborative Innovations for Health Promotion, within which this study was included, and the women were involved as participants in the research process.

The women’s health group defined long-term pain and mental issues as health issues they encountered in everyday life (20). Chronic pain is common in migrants with post-traumatic stress disorder (PTSD) (21, 22). In addition, an association with characteristics such as old age, being a woman, and experiencing living difficulties, was shown (21). Thus, promoting PA among migrant populations at risk of PTSD has been recommended, since PA has shown positive results with regard to lowering PTSD symptoms (23). Forced migrants and refugees are, however, groups difficult to engage in PA precisely due to the symptoms of PTSD, and other mental issues (23). Moreover, it has been emphasized that research among migrants in high-income settings has usually been conducted with a focus on communicable diseases or mental health, while the most common health issues among migrants are usually the same as among the host populations and therefore often disregarded (19). Thus, participatory research on health promotion taking into account health issues identified by the women’s health group, is important to explore.

Women are not a homogeneous group regarding health, and factors affecting health seem to be different between ethnic groups (24). Additionally, more mortality and morbidity occur in women with lower participation in the political arena and among those with lower economic autonomy (25). Also, lower mental quality of life, as well as lower self-esteem, is found among migrant women, which have been associated with their experiences of discrimination on three grounds, namely gender, social status, and ethnicity (26). When various social identities occur simultaneously, identities that have experienced oppression, health inequalities are complex (27), as among Moroccan migrant women, identifying themselves also as mothers and Muslims (8). Migrant women’s perspective would therefore be important to include in health promotion. Furthermore, besides being based on the heterogeneity of the migrant population, health information might also include social support. The social support may have a potential to promote health in counteracting the social isolation migrants might experience. And the combination of social support and health-promotive activities may possibly help change the collective social practice, which has previously been noted as a barrier to health promotion (10). Thus, to further enhance health promotion in a population of migrant women, innovative paths may be explored. The core values of participatory research (18) may lend themselves to this purpose in engaging a study population, in this case migrant women. Participatory research has been advantageous in health interventions among migrants, but little is explored regarding non-communicable diseases and health promotion (19). The aim of this study was therefore to describe how a collaborative women’s health initiative contributed to migrant women’s engagement in health promotion activities.

## Materials and methods

### Collaborative innovations for health promotion program

Based on CBPR approach a community health promotion program–Collaborative Innovations for Health Promotion was established in a socially disadvantaged neighborhood in Malmö (the third biggest city in Sweden) in year 2017. It was established by researchers from Malmö University together with the citizens from the neighborhood and stakeholders from public, private and non-profit organization sector (28). The overall aim of the program was to reduce health inequalities in socially deprived areas in Sweden through CBPR and community health promotion. As a first step toward the program future workshops were conducted in 2016 in the neighborhood. Here, citizens could express their needs and identify strategies to promote health. During the workshops, some citizens volunteered to become health promoters to help co-ordinate the activities within the program. Later they were employed part-time within the program. They also facilitated participant recruitment, and language interpretation. However, their most important function was to build trust between the researchers/stakeholders and the citizens. As a second step the research team, together with other stakeholders, and the health promoters created a CBPR model inspired by a model earlier developed by Wallerstein et al. (29) for planning collaboration and implementation of health promoting initiatives focusing on the problem areas earlier described. This resulted in that six health promoting co-creative labs were established. In the start of the program in 2016 women migrants participated in the future workshops that were conducted as an academic community partnership. In dialog between the community health promoting activities for women were discussed and the activities and research was decided from the women’s own perceived needs. Thus, the women were part of setting the research objectives in a planning process together with other stakeholder creating different sorts of health promotion labs in the overall program. The whole program was built upon sustainability as participation involved community members in all processes and they were actively involved from the planning phase. Thus, they were a part of building up the whole structure of the program together with the LHPs and researchers from the academia and other stakeholders. The LHPs developed their own function that created sustainability in the community through the CBPR program.

### Setting and design

The study was conducted in a city in the south of Sweden, in an area of the city regarded as one of the 16 particularly socially deprived areas in Sweden (30). A low belief in the future may be widespread among the population in such areas. This may be related to risk factors such as a higher degree of ill health and unemployment, and worse living conditions, or to global conflicts being present in the local environment, increasing disturbances. These populations may not be represented in the political system and public debates, and a low degree of trust between residents may impair the collective capacity (30, 31).

The program Collaborative Innovations for Health Promotion, within which this study was situated (32), used the CBPR approach and that approach was therefore also applied in this study. The program started as an initiative to counteract inequalities of health, and to promote health with innovative solutions (32). Two basic principles of CBPR are that “CBPR acknowledges community as a unit of identity” and that partnerships should be equitable (33). The membership in a community can be due to living in the same geographical area or due to other reasons for belonging to a group, such as an ethnic group, or to having a common interest, and multiple memberships may also occur (33). The belonging creates a social identity, in relation to others (33). To find communities connected to the issue under study is part of the CBPR approach and what should be built upon (33). The community of this study is connected by a geographical area and by a common interest to promote health in the local neighborhood. The local community became a partner in the program together with academia, the public sector, the private sector, as well as non-governmental organizations. They were all involved in the program’s steering committee, and together they discussed, tested, and implemented ideas of solutions for health promotion, based on the community members’ own needs (32). Building on what works in the community is another principle of CBPR (33). The strengths and resources may be identified at an early stage of the CBPR process (33). Identified health issues and populations became the focus for the health promotion work, such as women’s health, mental health, oral health, and social health. Lay health promoters (LHP), who were members of the community living in the area, were employed in the program. The LHPs had a central role operationalizing ideas and activities and gathering groups (34), and their role also included bridging between stakeholders and community, making reality understandable to all involved, in all directions (34). All LHPs were provided some training in PAR methodology (34). The training was not a fixed program more adapted to the LHPs needs as individual. One LHP became the facilitator for the women’s health group in this study. She also served as interpreter during activities and data collection. To gather groups, the LHP used short message service (SMS) and other communication means. Some of the women also needed a phone call prior to the activities. Women themselves also called the LHP when uncertain about times or places for activities. Flyers or posters were normally not distributed to communicate information. It was tested but not found useful. Usually, women heard about the activities from someone they knew and got access to the group through either the LHP or any of the other women already connected to the group.

This study had a qualitative design with a participatory approach. A main characteristic of participatory research is the circular iterative approach (18). This means that the process and evaluation can continue until clarification with the answers has been reached (18). Thus, dialog is needed and, accordingly, space for dialog was created and continuous dialogs were conducted with the women. Dialogs with the women’s health group started in October 2018 and conditions for health promotion were identified. Furthermore, during these dialogs, ideas of actions for health promotion were elaborated. Consequently, health-promotional activities were planned and initiated together with the women in November 2018 and continued during spring 2019. In April and May 2019, follow-up dialogs were conducted to create space for the women to reflect on their experiences of the activities.

The prior dialogs, conducted in 2018, were the basis for one study that aimed to identify conditions for health promotion together with women migrants in a Swedish context through a CBPR approach, and the results have been published elsewhere (20). Thus, the material for this second study was based on the follow-up dialogs conducted in 2019, with women who had participated in the health promotion activities, the study aim being to describe what contributes to the women’s engagement in these activities. With engagement means here their participation in health promotive activities with reflective participatory dialogs.

### Study participants

Information was presented for the women about data collection during preceding health promotion activities to invite them to participate. The LHP was in contact with those interested to participate prior to the day of data collection. All women participating in the dialogs had been taken part in the health promotion activities. Three story dialogs were completed in March–May 2019, including 8–9 women in each dialog. In total, 17 women participated in the dialogs. Nine of the women participated twice due to the iterative approach. The women were mainly migrants from Middle Eastern countries, the majority speaking Arabic, but also Persian occurred in the population. The time in Sweden varied; several had been in Sweden for quite some years and only a minority were newcomers. The majority did not have any work-related daily occupation. Most of the women got a monthly income from social security benefit or a pension, and a few from work or studies. In the perspective of intersectionality, the social identities of the study population may be identified as those that usually are deprived of privileges due to their gender and to belonging to an ethnic minority and having low socioeconomic status (27). Also, many of them had migrated from countries afflicted by armed conflicts, meaning that the population may be fulfilling another social identity, disability, due to mental issues because of traumas (35). Disability in the shape of stress and long-term pain had previously been acknowledged in the women’s health group (20).

### Health promotion activities

The health-promoting activities consisted of health circle meetings and PA sessions. Those activities were consequently the focus for the data collection of this study. But other activities were also offered within the major program, arranged by other actors. The Red Cross arranged courses in First Aid and riding a bicycle. A sewing group and a cook-a-long were going on, on a weekly basis. Gardening and parties were arranged occasionally, for example, to celebrate Eid al-Fitr and the international women’s day. The activities occurred in the daytime, as the women had required, both because they could find spare time then and because of the lower bus fare before 2 pm. The women included in this study had attended the health circle meetings or the PA sessions and most of them had attended both kinds of activities.

#### Physical activity sessions

The LHP led PA sessions twice a week for the women. The LHP had no formal PA education but had experience of participating in PA from before. The training sessions were free of charge, through the program, and open to all women. They normally lasted between 45 min and an hour and were located in a community youth center in the local neighborhood close to many of the women’s homes. The sessions took place in the daytime while no youths were present. Also, the physical environment was modified to make the women feel comfortable, for example, at the women’s request curtains were put up in the windows to limit visibility and the door was locked so that only the women could come into the hall. Furthermore, the PA sessions were adjusted to the women’s physical condition; for example, some could not stand up for a full hour, or at all, and they could then make the movements sitting on a chair. Therefore, everyone could attend on their own terms.

#### Health circle meetings

The health circle meetings took place during November 2018 until April 2019. First, after the initial story dialogs were completed in October 2018, a planning meeting was conducted. The LHP, the women, and the first author attended the meeting and together went through the subjects of the dialogs. The meeting was facilitated by the first author, who is a nurse trained in Sweden. Her prior knowledge about the healthcare system, and health in general, was advantageous when planning activities. However, ideas of guests were formed and discussed together. Then the first author offered and was assigned the task to contact and invite guests to the circle. Professionals in nearby healthcare centers were the first choice as guests, to engage the local community. Most of the professionals that were contacted were positive to the invitation and accepted it after discussion with their superior. The health circle was usually held once a week, with some interruptions for public holidays. The place for the meetings was a public space, set up by the municipality for the residents. The guests were usually sitting together with the group in a circle, with or without a power-point presentation. The purpose of this was to try to create more of a dialog and enable reflections between the women and the guest instead of a lecture with a speaker and an audience. Initially, the first author facilitated the health circle meetings, but later, after some meetings, she handed this role over to the LHP. This was made in accordance with the CBPR process, to make the activities more sustainable and promote the ownership of them among the community. Joint planning of the health circle meetings was a recurring element of the weekly meetings. In total, 15 health circle meetings were conducted with 4–17 attendees (see Table 1).

**Table 1:** The health circle meetings.

	Theme of the health circle meeting	Facilitator/guest	Participants
1	Planning	Researcher	5
2	Health literacy	Researcher	14
3	Stress	Researcher	7
4	Learning to grow sprouts	Lay health promoter	6–8
5	Basics physical activity and pain	Physiotherapists from a local healthcare center	9
6	Planning	Common activity	13
7	Writing questions to guests	Common activity	4
8	Information about activities in the area	The Red Cross	
9	Diabetes	Nurse specialized in diabetes from a local healthcare center	8
10	Depression	Nurse specialized in psychiatric care/Associate Professor from Malmö University	7
11	Food	Dietitian from a local healthcare center	14
12	Post-traumatic stress disorder	Counselor from the Red Cross	10
13	The Swedish healthcare system	Superior official from the regional healthcare organization	17
14	Dissatisfaction with the health care	The Patient Advisory Board	11
15	Sleep	Nurse specialized in psychiatric care from the municipality	10

### Data collection

Data was collected through the story-dialog method (36), a method that can be used in a CBPR approach program and may suit the population in this kind of setting (18). The story-dialog method comprises the participants’ stories, thus increasing the potential relevance for participants (36, 37). The study population for this study was formed around this group of migrant women in the women’s lab. Even if the story dialog was chosen by the researchers it was chosen from the women’s request to be able to have dialog meetings as a method in the lab. Accordingly, before the story dialog one of the women was asked to tell a story about what she has learned from the health promotion activities. The dialog and reflections then followed a structure according to the story-dialog method (36). Thus, all in the circle were invited to share reflections on what they felt about the story being told, and about similarities or differences compared to their own story. Following a structure is a support to deepen the story by means of what, why, and so what questions, and, lastly, to advance to actions, questions of a “now what” character are asked (36). Dialog is the core of participatory practice, and the Freirean pedagogy, as well as the creation of forums for dialog, is essential (37). To advance in the process of change, communication is needed, both by talking and, above all, by listening (37). Humans have various perceptions of truths and only through communication can we share and acknowledge differences (37). By acknowledging power structures between groups and being aware of the effect on relationships, one can, through dialog, equalize the power (37). In an equal dialog, both parties must acknowledge that they can learn from each other, and therefore respecting the other is a fundamental ability (37). Among those privileged in a dialog, a self-critical consciousness is important in order to understand the influence they have on those less privileged and what it encompasses (37). The balance of power in a dialog is very difficult to control, but as a privileged party in a dialog one can try to foster a habit of not having all the answers to questions (37). Thus, humility, as well as compassion, is central in dialog (37).

Data collection was conducted in the same localities as the health circle meetings, that is, localities that the women felt familiar with. Also, the same approach of sitting in a circle was used during data collection, to try to reduce power constructions and as a symbol that all the women were equal. Furthermore, the researchers did not have a pre-decided strategy for health promotion organized prior to the dialogs, as the CBPR approach involved not having the answers to what was needed to be done to promote health in this population. Instead, the strategy was developed together based on the skills in the community. The method also includes assigning tasks to all involved, a feature serving to increase participation in the dialog (36). Many of the women participated in the dialogs a second time since they attended the dialogs prior to the start of the health promotion activities. They were therefore well-prepared and could be involved in the tasks of the dialogs, for example, writing common notes.

When needed, the LHP translated the dialogs from Arabic into Swedish and vice versa. The dialogs were recorded for exact transcription. Two or three of the authors attended the dialogs each time, one or two as facilitators of the dialogs and one as observer and taking notes. Therefore, the authors are familiar with the material of the dialogs.

### Analysis

Thematic analysis according to Braun and Clarke was used to analyze the data from the transcribed story dialogs (38). The dialogs were listened to for familiarization and were then transcribed by the first author, according to phase one described by Braun and Clarke (38). Only the parts in Swedish were transcribed. The Arabic parts were not translated again during transcription. They had already been translated during the dialogs by the LHP into Swedish. The initial ideas found in this phase were about important factors as contributors to engagement in health promotion activities. In phase two the transcribed texts were imported in Nvivo qualitative data analysis software, in which the analysis was conducted (39). The text was manually coded in an inductive, data-driven way and on a more semantic level (38). In phase three, preliminary themes were formed by the codes, and the main themes were shaped. The important contributors were found in the main themes. The first draft of the analysis was discussed in the team of authors to check for accuracy of the themes. The themes were then refined and condensed from five to three themes, according to phase four described by Braun and Clarke (38). In this version of the analysis, the important contributors were found in all three main themes. During phase five of the analysis, the sub-themes and related texts were refined to fit the main themes.

### Ethical considerations

Oral and written information about the research of this study was given to the women prior to the data collection, to allow time for reflection and questions. The information was translated by the LHP into the women’s language. It was stressed that participation in the data collection was voluntary. Moreover, information that the women could still participate in the health promoting activities or withdraw at any time was emphasized. Written consent was obtained prior to the data collection. The Regional Ethical Committee in Lund (Reg. no. 2018/591) gave approval for this study.

## Results

Three contributors to engagement in health promotion activities were identified at an early stage of the analysis process, namely, the group, the facilitator, and place. They could at a later stage be connected to why they were important, that is, to how they motivated and supported the women and to how the dialog was conducted. This gave rise to the designated themes, which, together with the contributors, formed three main themes and nine sub-themes (see Table 2).

**Table 2:** Overview of contributors, themes, and sub-themes.

Contributors	The group	The facilitator	Place
Designated themes	*The group of women involved in the health promotion activities*	*The lay health promoter (LHP) as the facilitator*	*A physical place*
**The contributors motivated women to activity**	Social cohesion motivated women to start and continue activity	The LHP motivated women to join activities and to dare to move their bodies	Regular activities in a place generated motivation
**The socially supportive setting mattered for promoting health**	Activities supported social networking, which contributed to health promotion	The LHP as a supportive facilitator	Activities complemented existing health promotion offered in the local neighborhood
**Group dialog in place was advantageous for health knowledge acquisition**	Making sense and use of knowledge through group dialog	The LHP had an active dialog with the women	The health circle as a meeting point for increased health knowledge

### The contributors motivated women to activity

This theme included three sub-themes: “Social cohesion motivated women to start and continue activity”; “The LHP motivated women to join activities and to dare to move their bodies”; and “Regular activities in a place generated motivation”–referring to the group, the facilitator, and place, respectively, as contributors in relation to motivation.

The social cohesion was described by the women as a motivating factor to join the group and health-promoting activities. To get to know other women and have company was the most important reason to attend the activities. The women described it as difficult to conduct PA on one’s own, but when meeting with the group they were motivated to accomplish the training.

SD1: And at home I could not, when you are alone you cannot do training. Therefore, when with the group, that helps you. Because with the group you have feelings to…not feelings…that…motivate yes…to do everything.

Getting to know each other over time also motivated the women to continue with PA. Thus, when the women knew the other women’s health issues and knew that others also experienced pain, it was difficult to explain away one’s own absence from PA sessions due to pain. This was described by the women as internal competition that stimulated and motivated them to participate in PA.

SD1: The group, when knowing the others, for example, I see here that she has pain but exercises, how shall I…I also exercise. So, it will be this that one competes, one can say.

The internal competition was said to make behavior changes in relation to health more fun and may therefore have motivated health promotion within the group. Behavior changes were perceived by the women as difficult to sustain, but it was easier for them to uphold changes when they had regular meetings, because then they got new energy to continue the new behavior. The PA was perceived to decrease long-lasting pain, being therefore a way to handle pain in everyday life.

Moreover, the health promotion activities with the group motivated the women to convert health information into practice. Attending the group activities helped them make use of health knowledge they already had and make behavior changes in relation to health. Also, gaining knowledge in the health circle meetings had raised consciousness and confirmed knowledge from elsewhere.

SD3: … and some I already knew but I forget to use. That I, for example, want to take something from the floor maybe or carry something. I know how to do but I don’t use it and I still got pain in my back. But when I come here, I started to think more how I do even though I already knew, yes. How I can use such information.

Furthermore, the LHP motivated the women to attend the activities, and the women perceived that they needed someone to do that. There were no apparent obstacles, but getting started was difficult, initially requiring small steps, and the LHP had motivated them. One of the participants said that the activities had made a great difference in her life. Before, she felt as if she had no real life when she only worked and went home. Then she met the LHP and got motivated by her to start the activities within the project. She is now very grateful for this.

The women said that health information was obtained from both healthcare centers and the health promotion activities within this project. The information they received was similar, but within the project the LHP also worked to motivate the women. All these factors in combination were said to be useful. For example, the LHP motivated the women to dare move their bodies.

SD2: I try to motivate them as well. Even if you have pain issues, maybe you can try carefully, maybe.

Pain issues had hindered them, but when they got information from the healthcare and the health circle meetings as well as getting to practice and being motivated by the LHP, it felt secure to dare to move their bodies, which was found helpful. Previously, one woman said, it was difficult for her to bend her knees because of pain and therefore she could not do everyday activities. But because of daring to move and do the PA the pain had decreased. PA contributed to increased mobility and softness in the body among the women.

SD2: Yes, when I pray…when I pray, I couldn’t bend my knees…. Now I put a pillow and then bend. I feel a little pain but not as before. It’s not that much. I dare to do. Dare, yes!

Regular activities in an accessible meeting place generated motivation to continue with PA. To have a place to go, to get out of the house, was important for the women and helped them handle negative life situations. The women first joined the activities because of the social cohesion they offered. The women said it was easier to do PA together in a group, but the normal supply of group activities in the neighborhood was not accessible to them due to, for example, costly fees. It was thus important to have a specific place to motivate the women to gather for PA. However, after some time of conducting the PA, a habit was created. One woman said that the days when there was PA had become the most important days of the week.

SD3: I wait Tuesday and Thursday to meet with you and come to do physical activity. That is the most important day to me, Tuesday, and Thursday.

Thus, the habit to meet in the place assigned for PA created a need for PA, making the women want to continue to gather for PA in the community youth center. The PA, together with the health circle meetings, had formed a path to follow, one of the women said. The activities were described to have led to positive experiences, less pain and feeling strong enough to be able to carry things. A stop of the activities would mean going back to a situation with pain and stress. The regularity had created a need for PA which motivated the women to find time to continue the new habits. When one felt the need for the activities it was easier to come back to the path already entered on.

SD3: So that’s why I must continue with this. And even if I haven’t got so much time, I need to find time, I need to do everything just to continue and increase this. Now I can carry as well, that’s good, after training, so everything is connected. But I think, these things, if we shall go in for and make them regularly or just feel that we need them. It’s enough just to feel that I need them, then I correct myself and come back to this path.

### The socially supportive setting mattered for promoting health

This theme included three sub-themes: “Activities supported social networking, which contributed to health promotion”; “The LHP as a supportive facilitator”; and “Activities complemented existing health promotion offered in the local neighborhood”–referring to the group, the facilitator, and place, respectively, as contributors in relation to support.

The group activities supported social networking, which contributed to health promotion. The women described how they did not know each other before even though they lived close to each other. Meeting for the activities made it possible for them to get to know each other. Thus, the health-promotional activities supported the women in the process of forming a social network. When they met for PA twice a week, the focus was not only on the body, but also on the company. The PA sessions developed, and after some time the last 15 min of the sessions were used for dancing, which created a lot of laughter.

SD1: When we come for physical activity it’s not only…it’s not only about the body, it’s about company and [being] social. Yes, it’s exactly like last time, we were, we were laughing a lot. During the last 15 minutes we danced.

One woman had noticed that the sessions had a calming effect and that she fell asleep easier in the evenings. Another woman expressed her gratitude for the knowledge and company she had gained from the activities, and yet another woman agreed that the PA sessions had been important to avoid loneliness. The women talked about illnesses and health issues that had affected them, but these issues were described as a natural part of life. Nonetheless, the women emphasized the importance of dispelling one’s thoughts and of meeting with other people so as not to be alone during periods of ill health.

SD1: Yes, but it’s normal in life to get pain and have many health problems. But it’s great that I get out to meet others and that’s better than to sit and think only about my health problems.

The company of the group was also important for the women when exploring new activities. The women said that the feeling of having company also contributed to increased courage, so that they dared to ask questions to the guests visiting the group during health circle meetings.

The LHP was perceived by the women as a supportive facilitator. She supported the women in finding and participating in activities they were interested in, both within and outside the neighborhood and the program. These activities helped the women develop and they were therefore grateful to the LHP and all who had been involved in the development of activities. Both the women and the LHP perceived that they needed someone to support them when planning to explore new activities, someone to take the lead for travel arrangements. The women perceived it as safe to attend the health-promotional activities because they knew the LHP from before and trusted her, which was important. One of the women said that a good person was the one to collect women in the neighborhood for the purpose of the health promotion activities, and therefore the LHP was regarded by the women as a good leader.

SD2: You as…I don’t know…/…/ when you want to do something, circle here in the area. We take good person here to gather other women. So, they also, you the same know T [the LHP].

The LHP focused individually on them all, seeing each of them, which was considered important. The women said that she taught them a lot during PA. She was, for example, teaching breathing and was observant of them individually, making sure they were breathing correctly. This helped and the breathing got better. The LHP was supportive also outside the health-promotional activities of the project. She helped solve the women’s issues, and since they had similar issues as herself, she also benefited when helping them.

SD3: Yes, in some cases I need to help myself [with] almost the same issue or problem. No, I think I help them, it gets easier to help myself.

The women said that it helped to learn about activities within the local neighborhood in order to get support in the local area. Furthermore, in the health circle meetings, they met with guests who were professionals, and there they dared to ask questions, since they were in a safe place, feeling comfortable and supported by the group and the LHP. Thus, receiving health information in the health circle meetings was perceived as better than to obtain it in the healthcare center.

In contrast, the previous activities offered in the local area did not support health promotion among the women. Thus, desired activities, such as swimming, were available in a nearby area but could normally not be used by the women due to gender issues. Women’s-only time was seldomly available, and if it was, the entrance fee was expensive. The healthcare center setting was not supportive of the women either, the communication there not being adjusted to their needs. They perceived the setting as stressful and felt that the physicians did not have time for them, which made the women avoid asking questions about their health issues.

SD3: Yes, the healthcare center [has] only limited time. We can’t ask and get answers and we feel that the physician is also stressed.

However, disagreement existed regarding support from healthcare centers, where some had gotten help, and some had not. The PA sessions in the project were compared to the physiotherapy in healthcare centers, similar successful, as well as better, results having been reached. The whole body was in focus during PA, while only parts were in focus in healthcare centers. The PA was very much appreciated and said by the women to be needed, contributing to make a difference, and the women therefore wished that it be continued. Moreover, the activities were supportive for the women in accomplishing other goals as well. One of the women got help with breathing and balance during PA, which helped her learn how to ride a bike, a course that was offered within the larger program.

### Group dialog in place was advantageous for health knowledge acquisition

This theme included three sub-themes: “Making sense and use of knowledge through group dialog”; “The LHP had an active dialog with the women”; and “The health circle as a meeting point for increased health knowledge”–referring to the group, the facilitator, and place, respectively, as contributors in relation to dialog.

Dialog in the group was said to have provided an opportunity for the women to obtain health-promotive information, not only from the guests, but also from each other when listening to the others’ stories. It was helpful to hear about others’ situations and ideas. Moreover, thanks to the dialog, one could find words to describe one’s own situation. As it was sometimes difficult to describe feelings, someone else’s story could be useful in the creation of one’s own.

SD3: Maybe one feels something but cannot describe [it] herself maybe. Someone else describes their situation or… yes, one can get information from others as well, not just specialists.

The dialog in the group was described by the women to have increased the understanding of the information they had received during health circle meetings. Moreover, most of the guests in the health circle meetings spoke Swedish, which was translated into Arabic. The women could ask their questions and conduct the dialog in their own language, which was then translated into Swedish. Even though the dialog was translated, the women perceived that learning Swedish was promoted. In the city where the women lived, daily dialogs in many stores, for example, could be held in their own language. It was therefore important, they said, to have the group dialogs in Swedish to develop the language.

The dialog about behavioral changes in relation to health in the health circle meetings encouraged health promotion. The information they received was not always new to them, but to have a forum in which to share achieved behavior changes was described to have helped the women change.

SD3: Okay, but we already know how dangerous it is to eat deep fried foods or fat and sugar. But we get careless sometimes when we don’t have someone to remind us all the time. We try not to care and eat as we like. But now when we have started the health circle, we feel that there’s someone, we will tell someone that we have changed something.

The dialog between the women continued after the common activities finished, as they discussed on the phone what they had learned during activities. The women also communicated what they learned to others outside the group. Thus, they used the information themselves and then spread it to others–their children, grandchildren, cousins, and friends.

Furthermore, the use of dialog in the group helped them formulate common plans. For example, collaboration in the community was suggested, to fight powerlessness and discrimination and grow strong together. The group described healthcare dissatisfaction, which they wanted to complain about. They specifically pointed out that they dared to speak out about their dissatisfaction but that as migrants they were not listened to when they did, and that they therefore perceived their rights as different compared to Swedes.

SD2: We as migrants, if we complain…one doesn’t take it seriously when migrants complain.

The LHP had an active dialog with the women. The women described how the LHP had invited them to join the group and the health promotion activities and that she used her social network when doing so. The LHP communicated actively with the group and worked hard to remind them about the activities. They had the information and knew the schedule of the activities, but they still, according to both the LHP and the women, needed someone to remind them. Being active in reminding participants was regarded as essential for being a good facilitator. The women said that the LHP had worked hard for them, and that she was kind and had patience with them all, even though they were all different. The LHP had tried to accommodate to and have patience with all women since they had different ways of thinking, behaviors, traditions, and dialects. She tried to answer their questions and adjust to the women’s wishes, but sometimes it was not possible to do everything right. Consequently, the LHP had a large workload.

SD1: But I didn’t agree with all women about a time. And I must divide [my time] between many and adjust with all. But therefore, I couldn’t do.

In the health circle meetings, the women met physically in a place assigned for dialog where they could help each other understand information from guests. The health information they received was relevant for them and helpful because it was needed right now. Furthermore, health information on the internet was perceived as complicated, both to find and to get through. The communication of health information in the health circle was therefore perceived as much easier, enabling them to obtain correct, simple, and summarized information.

SD3: Because the net [internet] is very complicated. One cannot get in, not all are good on the net. I do not like the net, I think it’s very troublesome…, that what I want, one must go through whole pages and sometimes one ends up on the wrong page. So that’s because it gets a lot to some people. Easier to just go through discussion where one receives correct, simple, concluded information.

To meet physically in a social place was shown to be advantageous for the women. One woman who had issues with memory loss described how meeting face to face had helped her brain become more active, which had made her remember better. To meet people of a similar age and in a similar situation helped to renew and change thinking.

The women perceived that health knowledge was needed to promote their own health. To develop health knowledge and obtain new information, dialog was needed. It was not only a matter of knowing about healthy foods but also about the ability to evaluate the correctness of physicians’ prescriptions. The ability to read information about a prescribed medicine or trust the physician was a balance one needed to handle to avoid serious side effects. One woman said that the program in the neighborhood and the health circle meetings had supported this process to gain health knowledge.

SD2: Yes, I have new information from that circle, I didn’t know much.

The women shared that all the information communicated by the guests in the health circle meetings was important and had been useful. The themes that were addressed were, *inter alia*, diabetes, pain, mental issues, and the stress that many of the women were experiencing. Many examples of how they had been helped by what they had learned were brought up in the dialogs. However, even though they thought the information was useful and important, they would have wanted the information in writing too, in their own language. The double communication would help them to be reminded of the information and would be helpful if they had missed a health circle meeting.

SD3: We get useful information from here, from the visitors. But we need brochure in our language, Arabic, to remind ourselves.

## Discussion

This study aimed to describe how a collaborative women’s health initiative contributed to migrant women’s engagement in health promotion activities. The findings of the study show that being a group of women, having the LHP as facilitator, and having a place to meet, all contributed to the migrant women’s engagement in health-promotive activities. Processes of positive changes were started and continued through motivation, support, and dialog where the perspective of the women was considered, and participation promoted.

Women in this study described how the health-promoting activities contributed to stress relief as well as to decreased pain. A key implication was that support by the group was important for accessing health promotion activities. Thus, social cohesion motivated the women both to join and to continue activities. The activities thus helped to form social networks. The comorbidity of physical and mental issues has previously been described among migrants coming from countries afflicted by armed conflicts (22, 23). Previous research has also shown that low social support among migrants was associated with a higher degree of pain (40), which might indicate that an increase in social support may affect pain positively. Furthermore, previous research has shown that around 55% of Syrian newly arrived women migrants had a weak social support in Sweden (41). Also, research has shown that around 20% of newly arrived migrants from Syria felt excluded and isolated in the Swedish society, and this was associated with mental issues like PTSD, anxiety, depression, and low subjective well-being (35). Social support can be seen as a resource, contributing to a person’s ability to handle stressors in everyday life (42). Moreover, Sjögren Forss et al. suggest that newly arrived migrants should be informed about the benefits of PA in promoting mental well-being, and that a multisectoral approach may be needed to promote PA (12). Thus, the possible existing difficulties for migrants to engage in PA in order to promote health must be considered (23). This is in accordance with the results of this study, where health information was complemented by additional factors, such as the social cohesion, which facilitated the engagement in health-promotional activities.

Another key implication was that, in the process of learning, the group played a role for the women, because they helped each other describe their situation. They also assisted each other in making the information they received understandable, and, strengthened by the support they gave each other, they dared to ask questions to the guests visiting the group. This implies that the women as a group were empowered. As described by Freire, dialogs and reflections in a social group can contribute to empowerment processes and social justice (16). Migrant women have previously scored lower in measures of self-esteem compared to non-migrants, which has been suggested to have an association with the triple discrimination they might experience (26). The women’s experiences of discrimination and powerlessness when trying to make their voices heard in the health care, were also acknowledged in this study. However, with the help of dialog in the group they were able to start to formulate ideas for a common path forward.

The LHP contributed to the women’s health-promotional process through motivation and support. Improvements such as better breathing and daring to move one’s body were initiated by previous or new knowledge from the health circle and health care. A key implication was then that the women could make use of their knowledge when motivated and supported by the LHP. They could practice and get corrections individually within the group along the way. The relation between the LHP and the women have similarities with peer-supporters. Peer-supporters have previously been used among women in situations of for example breast feeding, and among migrants in a Swedish context (43, 44). Such a role has been found rewarding for the one being in a position with ability to support others, and helpful for those in need of support (43). The flexibility of the role of the peer-supporter was highlighted as advantageous, thus, instead of having a fixed program for all, the peer-supporter could support the individual as everyone are different (44). Kåks et al. includes also how the peer-supporter role function in bridging between the host country and the previous home country and thus the importance in the process of empowerment (44). However, peer supporter’s role is often to bridge between the health sectors/social sectors and the community. LHP is more of advocates for the community and are not parts of the health care system (32).

Another implication was the LHP’s very active dialog with the migrant women–a group sometimes referred to as a hard-to-reach population (45)–which motivated them to participate. The participatory approach to research is important in order for the participants to be able to raise their voices and, by extension, for the knowledge production. Situated knowledge is about knowledge being produced in relation to power balances, connected to gender, class, ethnicity, etc., which means that the social identities of a person are involved in the knowledge production (46). Thus, various perspectives are needed for a better comprehensive understanding of reality (46). The approach of this study has its roots in CBPR and the teachings of Freire, where the opposite of hierarchy is advocated (47) and where the aim is to include and distribute the power to those that the research is about, in order to generate social justice and health equity (47). As Suarez-Balcazar et al. point out, difficulties may arise when incorporating marginalized populations in the research process (47). A way for participation can then be a forum where the population may feel trust (47). This was acknowledged in this study, where the LHP was regarded as trustworthy and as a good person, something which was perceived as needed for gathering the women. A previous study in the program where this study was included, highlights that trust was important within the program and therefore worked with from the start (34). The LHPs included in the program contributed to establishing trust between the community and the other partners due to having a constant dialog regarding needs and facilitating the community members’ voices (34). Having a dialog and working with inclusion and power relations within the group was not achieved without friction, though. As the LHP in the present study said, she tried to adjust to all the women but sometimes it was not enough. Different formations of individuals and groups occurred within the group. It took a lot of time for the LHP to try to include all the women and their wishes regarding the activities. In the overall program all community members were involved in focus group session where they discuss collaboration and partnership related to culture and power in the labs every 6 month (32). LHPs was also trained in conflict methods with professional mentors from social care and Save the children. If a problem could not be solved in the group, the LHP had a possibility to discuss this with the professionals in the overall program. Various strategies have been tested to provide the LHPs with tools for own use in conflict situation. Story dialog method was such a tool and often used in the overall program, as a conflict method. Since stories often involves dilemmas or conflicts, the reflections in the group dialog becomes an important part of solving conflicts. Furthermore, it was shared during dialogs that the women needed someone to take the lead when discovering new activities and that the LHP was a suitable person for doing this, thus supporting a hierarchical structure of the group. A hierarchical structure is not compatible with the participatory approach and not something to aim for. However, in this group such a structure was regarded as needed to initiate engagement in the group and activities. Another interesting piece of information found in this material was that the inclusion of women happened based on the LHP’s outreaching capacity. First, the LHP used her social network to invite women, and then she invited women that she happened to come across during everyday life. This was great for those women who were part of her network or who moved in her vicinity, but others may have been excluded. Thus, although this is a way to reach populations that are otherwise hard to reach, there is no control of what persons are excluded.

Place was found to be a contributor to positive changes in health promotion among the women. The health circle, where the women met in a physical social place, supported the process to gain health knowledge. The women helped each other understand the health information, and they preferred obtaining summarized health information to finding information on the internet. A key implication was that the health-promotional activities in this study gave an opportunity to meet and to have a dialog with professionals in a calmer place, contributing to giving space to the women’s voices and questions. In the stressful environment within health care, they did not ask the questions they wanted. The issue of power balance may also be important in this perspective. The power balance between healthcare personnel and patients in general has previously been described as patients having a threefold subordination: the institutional, the existential, and the cognitive (48). This means that they are lowest in the institution hierarchy, vulnerable due to disease, and least knowledgeable compared to the personnel they meet in the health care (48). In the last decade there has been a focus on person-centered care in Sweden, aiming to include the perspective of the patients more. However, evaluations of the implementation show that patients belonging to the general population in Sweden are not satisfied with the care they get (49). Adding the perspective of intersectionality, a population such as the one focused on in the present study may feel discriminated against due to being women and belonging to a minority ethnic group (27). The calmer places that the women referred to were the common spaces within the neighborhood that were used for the activities within the program (34). These were facilitated by the municipality of Malmö, welcoming all and empowering citizens to participate (34). The women were therefore familiar with the meeting points for the activities. Places like this have previously been described as third places, places that are neither home nor workplaces (50). They were necessary in the program that this study is based on, for the inclusion of people (34). According to previous research, third places are important in a migrant population in that they can serve as places for retreat (51) and also as places where identities prior to and other than the identity of being a migrant could be elaborated (51). Dialog in a calm place therefore seems to counteract subordination and feelings of discrimination, instead enhancing conditions for acquiring health knowledge.

The main implication of this study was that the women made use of their health knowledge and put it into practice through the group, the facilitator, and place. By continuous participation and reflection in both the health-circles and in physical activity they collectively got more education about the importance of physical activity, both in theory and in practice. As in all action research, the knowledge acquisition took place by situated learning, (“learning by doing”) as the women learned by actively participating in both physical activity as well as in dialog-based health circle meetings. They expressed a strong wish to share their knowledge with each other and as a result, they felt that they had the opportunity to affect their situation. By learning from each other they realized that they also could have a stronger influence in the society and that their knowledge also was important. This points toward a situation of critical health literacy (5). This level of health literacy also includes the level called interactive health literacy, where space is created for reflection and dialog around one’s own health situation (5). Through interaction, autonomy and empowerment can develop (7). The level of critical health literacy means that the individual not only reflects critically regarding health information but also puts the knowledge into practice, both for themselves and for others in the group/community (5). Usually within health care, health information is a one-way communication, not including a dialog with the patient, and this may be referred to as a functional level of health literacy (5). A similar situation was described by the women in this study where they did not ask questions regarding their health issues in the healthcare centers. Instead, another place was needed, with a focus on reflections and dialogs and where they got support from each other and the LHP, and together created a calmer place. The women described how having a place to meet regularly with women with similar health issues supported them to engage in the health-promotional activities. Those activities may therefore be conducive to increased control of the process of one’s own health, in other words to an increase of empowerment.

### Strengths and limitations

One limitation of this study is that the LHP who facilitated the health-promotive activities was also the facilitator of the story dialogs, where accounts of the women’s experiences of the activities were collected. Very few negative accounts were shared, which could possibly be due to loyalty to the LHP. Nevertheless, the women participating in the women’s health group were all part of the development of the activities and a collaborative unit. Therefore, including the LHP in the dialogs made for a bridge between the women and the researchers, thus promoting the accounts.

In relation to this, one could see the language barrier as a limitation, as the translations between Arabic and Swedish were not accomplished by a certified translator. In Sweden, persons have a legal right to have access to an interpreter when communicating with institutions. However, a professional interpreter does not, by default, secure a problem-free translation (52), and since this study was completed outside a healthcare setting another kind of interpreter could be useful. The LHP who translated the dialogs was a member of the community and was perfectly placed to seize what was going on within the group, on an everyday basis. As she knew the women and had gained their trust and had a more contextual understanding of the language, one may argue that she could perform a better translation of the women’s accounts. The value of this position for the program and the research was immense. And if there has been any violation of the translations, it has been done in order to better capture the semantics of the dialogs.

Another limitation of this study was that the participants were not part of the analysis process. When using the CBPR approach, participants should be included in the whole research process, thus also in the analysis. A continuous process between the researcher and the participants could secure that the perspective of the participants has been grasped, thus limiting the risk of the result being influenced by the researchers’ bias and own assumptions (53). The second level synthesis is an analysis method that can be used in combination with the story-dialog method, including the participants in the analysis process (36). However, since the three story-dialogs covered by this study needed to be conducted on different days, the overall analysis was not completed together with the women. Therefore, thematic analysis was chosen instead, offering a straightforward approach when wanting to stay close to the data (38). To try to minimize the risk of bias, the analysis was completed close to the text, where few abstractions were made, which may be regarded as a realist approach (38). Also, most of the researchers were familiar with the material, having participated during the dialogs, and therefore functioned as a check for accuracy during the analysis process. When the analysis is not conducted together with the participants, one way to achieve validity is to bring back the result of the analysis to the participants, although different opinions have been declared regarding this (53). Accordingly, the results were taken back to the community where the study was conducted, three and a half years after the data was collected. Due to the Covid pandemic and other interruptions of the group and activities, the same study population could not be approached. However, women’s health groups were still active or had started anew within the community, with both new and old participants. They could confirm the results, which also served as a background to the continuation of the iterative process.

## Conclusion

The overarching implication of this study was that the women made use of their health knowledge and put it into practice. The group, the facilitator, and place were all contributors that, in combination, achieved this. What this study adds is that it focuses not only on social cohesion in the group and on the lay health promoter as facilitator but also on place as a contributor to positive change. Motivation, support, and dialog were important themes in the realization of the activities. The support by the group, the facilitator’s ability to motivate the women, and regularly having dialogs in a meeting place were thus three other key implications of the health-promotional activities. Based on these implications, health education may develop from health information delivery on a level of functional health literacy to a level of critical health literacy, where the health knowledge is put into practice.

### Future research

The accounts of the women were very positive about how the health promotion activities had contributed to their engagement in the promotion of their own health. The two studies concerning the women’s health group that have been conducted within the Collaborative Innovations for Health Promotion program, focus on what the women themselves can do to promote their health. As a continuation of the iterative process, it would be valuable to research accounts of health care professionals and other practitioners in relation to health promotion among migrant women, regarding the women’s needs of support from others to promote their health and how they can collaborate with the health care. Further research should also explore in what institution these kinds of activities should be facilitated in a Swedish context. It is not, in a traditional perspective, a suitable task for the Swedish health care, nor for the social security care, but should not be driven on a voluntary basis.

## Data availability statement

The raw data supporting the conclusions of this article will be made available by the authors, without undue reservation.

## Ethics statement

The studies involving human participants were reviewed and approved by the Regional Ethical Committee in Lund. The patients/participants provided their written informed consent to participate in this study.

## Author contributions

CL, KSF, CK, AK, and MR were involved in the design of the study and in the data collection. CL was performed overall analysis under guidance of the other authors. CL wrote the manuscript in close collaboration with the other authors. All authors contributed to the article and approved the submitted version.

## Funding

The programme where this study was included was funded by VINNOVA (Reg. no. 2016-00421, 2017-01272), primarily for the establishment of the health-promoting platform (and not for research conducted within this platform).

## Conflict of interest

The authors declare that the research was conducted in the absence of any commercial or financial relationships that could be construed as a potential conflict of interest.

## Publisher’s note

All claims expressed in this article are solely those of the authors and do not necessarily represent those of their affiliated organizations, or those of the publisher, the editors and the reviewers. Any product that may be evaluated in this article, or claim that may be made by its manufacturer, is not guaranteed or endorsed by the publisher.

## Abbreviations

CBPR: community-based participatory research
LHP: lay health promoters
PA: physical activity
PTSD: post-traumatic stress disorder
SMS: short message service
WHO: World Health Organization
