# Supplementary material for: Migrant women’s engagement in health-promotive activities through a women’s health collaboration
Source: Front Public Health. 2023 Jun 15;11:1106972. doi: 10.3389/fpubh.2023.1106972 (PMC10308282; doi:10.3389/fpubh.2023.1106972)
Supplement: Supplementary file 1 [file Data_Sheet_1.docx]

**Supplementary material**

Table 3: Coding tree

| **Meaning unit** | **Code** | **Sub-theme** | **Theme** |
| --- | --- | --- | --- |
| And at home I couldn’t, when you’re alone you can’t exercise. Therefore, when you’re with the group, it helps. Because when you’re with the group you have feelings to… not feelings…that…motivate yes… to do everything. | Motivating to engage in physical activity in a group | Social cohesion motivated women to start and continue activity | The contributors motivated women to activity |
| There are no obstacles that prevent them from getting out, but they have no desire to do [so], they need someone to motivate them. | Participants need someone to motivate them | The LHP motivated women to join activities and to dare to move their bodies |  |
| And second, I got used to it so after the weekend the time comes when I feel I need to move, I need to do this. So then these same movements come. | The PA habit has created a need for PA | Regular activities in a place generated motivation |  |
| We didn’t know each other before. During that time, we have possibility to meet all together and recognize each other. | We didn’t know each other before | Activities supported social networking, which contributed to health promotion | The socially supportive setting mattered for promoting health |
| During PA she looks at us, at each one, how she breathes. She tells someone who’s not breathing correctly: breathe like that. So, she taught them.  She focuses with all.  Focuses, yes. | The leader focused individually on us all | The LHP as a supportive facilitator |  |
| Exactly, they feel more comfortable here when they meet specialists to talk to. That time they don’t get in the healthcare center. | The participants felt safer to talk to specialists in their own environment | Activities complemented existing health promotion offered in the local neighborhood |  |
| Yes, if dialogue on subject, better for understanding than just to say.  What did you say? Than just?  Than just reading from a paper, dialogue better. Everything and we can understand.  One discusses issue oneself with specialists, maybe one gets more information, even better than if the doctor only gives advice without discussion between. | The dialogue in the group increases understanding in the group | Making sense and use of knowledge through group dialogue | Group dialogue in place was advantageous for health knowledge aquisition |
| And they actually need, not me…(?), not that curious. I try to adjust to them all the time and answer all their questions maybe or what they think about. | The leader has tried to adjust to the participants | The LHP had an active dialogue with the women |  |
| Okay, absolutely. And how do we get health knowledge?  What I think [is] number one [is] when we are social. But also, as you are doing now in the area, get information from others, from you who have project.  From dialogue we can get new information.  Yes, myself I have new information from that circle, I didn’t know much  You feel you got information from the health circle?  Yes, yes. | Dialogue contributed to improved health knowledge | The health circle as a meeting point for increased health knowledge |  |
